# Identification and Characterization of the miRNA Transcriptome Controlling Green Pigmentation of Chicken Eggshells

**DOI:** 10.3390/genes15060811

**Published:** 2024-06-19

**Authors:** Kai Shi, Dongfeng Li, Xusheng Jiang, Yuesong Du, Minli Yu

**Affiliations:** College of Animal Science and Technology, Nanjing Agricultural University, Nanjing 210095, China; 2020205003@stu.njau.edu.cn (K.S.); lidongfeng@njau.edu.cn (D.L.);

**Keywords:** miRNAs, eggshell color, hens, egg quality

## Abstract

Green eggs are mainly caused by inserting an avian endogenous retrovirus (EVA-HP) fragment into the *SLCO1B3* gene. Although the genotypes for this insertion allele are consistent, eggshell color (ESC) may vary after a peak laying period; light-colored eggs are undesired by consumers and farmers and result in financial loss, so it is necessary to resolve this problem. miRNAs are small non-coding RNAs that exert essential functions in animal development and diseases. However, the regulatory miRNAs and detailed molecular mechanisms regulating eggshell greenness remain unclear. In the present study, we determined the genotype of green-eggshell hens through the detection of a homozygous allele insertion in the *SLCO1B3* gene. The shell gland epithelium was obtained from green-eggshell hens that produced white and green shell eggs to perform transcriptome sequencing and investigate the important regulatory mechanisms that influence the ESC. Approximately 921 miRNAs were expressed in these two groups, which included 587 known miRNAs and 334 novel miRNAs, among which 44 were differentially expressed. There were 22 miRNAs that were significantly upregulated in the green and white groups, respectively, which targeted hundreds of genes, including *KIT*, *HMOX2*, and several solute carrier family genes. A Gene Ontology enrichment analysis of the target genes showed that the differentially expressed miRNA-targeted genes mainly belonged to the functional categories of homophilic cell adhesion, gland development, the Wnt signaling pathway, and epithelial tube morphogenesis. A KEGG enrichment analysis showed that the Hedgehog signaling pathway was significantly transformed in this study. The current study provides an overview of the miRNA expression profiles and the interaction between the miRNAs and their target genes. It provides valuable insights into the molecular mechanisms underlying green eggshell pigmentation, screening more effective hens to produce stable green eggs and obtaining higher economic benefits.

## 1. Introduction

Eggshell color (ESC) is an important economic trait that is affected by various factors such as genes, health status, and nutrition [1]. White and brown are common ESCs in poultry and are, respectively, preferred in North America and Asia [2]. A blue ESC can also be found in several breeds, such as Araucanas and Dongxiang chicken, which result from biliverdin accumulation [3,4]. Blue eggs are preferred for their better performance, taste, and quality than when compared to white eggs [5]. Green eggs are mainly caused by the insertion of an avian endogenous retrovirus (EVA-HP) fragment into the *SLCO1B3* gene, and individuals homozygous and heterozygous for this insertion have green shells [6]. It has also been reported that two cis-regulatory single nucleotide polymorphism (SNP) variants upstream of *ABCG2* could cause green duck eggshells [7], and others determined an additional SNP in the promoter of *ABCG2* can cause an identical phenotype [8]. Although chickens have the same genotype, the ESC may vary from dark to light blue after a peak laying period, resulting in severe financial loss. Further studies suggest that lncRNAs could regulate color formation, immunity, and lipid metabolism to change the darkness of blue eggshells [9]. This study suggests that other ncRNAs may exert similar functions in pigmentation.

MicroRNAs (miRNAs) pertain to a class of small (18–24 nucleotides), endogenous, non-coding RNAs that bind to the 3′ UTR of their target mRNA to repress protein translation or directly degrade mRNA by forming the RNA-induced silencing complex [10,11]. Growing evidence has demonstrated that miRNAs play important roles during cell growth, differentiation, pathogenesis, and disease prevention [12,13]. A previous study has shown that the compounds biliverdin and biliverdin IX zinc chelate are present in green eggshells; in contrast, protoporphyrin has been identified in brown and light brown eggshells [14]. In addition, miR-144-3p, which belongs to the miR-144 family, is upregulated in the shell glands of green-shelled eggs, suggesting that miR-144 is involved in pigment formation [15]. Recent studies have suggested that miR-144 and miR-451 can accelerate the differentiation of hematopoietic stem cells and hinder their growth [16]. These findings suggest that miRNAs might play a regulatory role in the ESC. Most studies have focused on the formation of brown eggshells and their regulatory mechanisms [17,18]. However, studies regarding the genes that regulate other colors of eggshells such as green are limited. In addition, the underlying genetic mechanisms of the ESC remain unclear. Transcriptome analysis with deep sequencing technology is a major platform for discovering functional genes that regulate biological functions at the miRNA level [19,20].

In this study, we used the Solexa deep sequencing approach to investigate the regulatory mechanisms behind changes in green and white eggshells from homozygous green-eggshell hens. We identified a total of 44 differentially expressed miRNAs, and found that their target genes are involved in gland development, the Wnt signaling pathway, epithelial tube morphogenesis, and the Hedgehog signaling pathway. These findings offer a new understanding of how eggshell color forms and hold promising potential for driving industry development in the future.

## 2. Materials and Methods

### 2.1. Chickens and Sample Collection

A total of 1137 pure-line 53-week-old Suqin laying hens (Tianmu Ltd., Changzhou, Jiangsu, China) that lay green eggs were used in this study. All the following procedures were strictly performed according to the regulations and guidelines established by the Nanjing Agricultural University Animal Care and Use Committee. According to previous methods [1], the chickens were genotyped for the insertion of the EVA-HP fragment. We used a DNA extraction Kit (DP304, Tiangen Ltd., Beijing, China) to extract chicken DNA, which was then amplified using three primers (F1: GCATTTCACAAACGGGTGTA; F2: CCCAGCAGTAAGCCCTACAT; R: CAAAACCACAAAGGTAATGTTCA). Homozygous-eggshell hens produced a 190 bp amplified product, heterozygous-eggshell hens showed 190 bp and 345 bp bands, and non-green-eggshell chickens displayed a 345 bp product. Eight hundred and ninety-three (893) chickens homozygous for the insertion were selected for the subsequent step.

At 53 weeks of age, eggs were collected on three successive days. ESC, eggshell weight (ESW), eggshell strength (ESS), eggshell thickness (EST), and egg shape index (ESI) were measured according to a previous study [1]. By using a colorimeter (3NH-NR110, 3NH, Shenzhen, Guangdong, China) to compare the eggshell color, 16 hens that lay green eggs and 17 that lay white eggs were selected. The two groups significantly differed in ESC, but not in terms of other eggshell properties. Three individuals in each group were randomly selected and sacrificed 6 h before oviposition, and the shell gland epithelium was collected and flash-frozen in liquid nitrogen [1]. The group data are presented in Table 1.

### 2.2. RNA Preparation

Total RNA of the shell gland epithelium was extracted separately by the Trizol^®^ technique (Trizol^®^ group) (Invitrogen, Carlsbad, CA, USA) in accordance with manufacturer’s instructions, including homogenization, phase separation, RNA precipitation, washing, resuspension, and RNA dissolving. The quality of the total RNA was assessed via routine agarose gel analysis, while the concentration was determined using spectrophotometry (ND-1000, Thermo Scientific, Waltham, MA, USA). Once extracted, the RNA was stored at −80 °C.

### 2.3. High-Throughput Sequencing

MicroRNA deep sequencing was performed on an Illumina HiSeq 2500 analyzer, facilitated by Biomarker Technologies Corporation in Beijing, China. We used approximately 2.5 μg of RNA per sample for RNA preparations. The sequencing libraries were constructed using the NEBNext Ultra II Small RNA Sample Library Prep Kit from (New England Biolabs, Ipswich, MA, USA), following the prescribed guidelines. Index codes were added to link sequences to their respective samples. The library construction included several steps: (1) isolation of RNA fragments, 10 to 40 nucleotides in length, via 15% denaturing polyacrylamide gel electrophoresis; (2) ligation with 5′ and 3′ adaptors, followed by reverse transcription of the short RNAs into cDNA based on Illumina’s protocol; (3) purification of PCR products with the AMPure XP system (Agencourt Bioscience Corporation, Beverly, MA, USA), with library quality assessed using the Agilent Bioanalyzer 2100 system (Agilent Technologies, Palo Alto, CA, USA); (4) after cluster generation, sequencing was carried out on the same Illumina platform, yielding paired-end reads.

The raw sequences were first processed using Illumina’s Genome Analyzer Pipeline software (v2.0.5) to remove adapter sequences, along with low-quality and low-copy sequences. Small RNA sequences, 18 to 30 nucleotides in length, were then extracted and filtered using the mRNA, RFam, and Repbase databases. Simultaneously, metrics such as Q20 and Q30 scores, GC content, and sequence duplication levels of the cleaned reads were calculated. These unique sequences were compared against the miRNA database (miRBase 20.0) via a BLASTn search to identify conserved miRNAs in chickens (http://www.mirbase.org/ assessed on 10 February 2022) assessed on 10 February 2022. To identify potential miRNA precursor sequences, a maximum of three mismatches was permitted between the short identified miRNAs and known animal miRNAs. Further, all identified mature miRNA sequences underwent BLAST analysis against the chicken genome sequences available on the Ensembl database (https://ftp.ensembl.org/pub/release-92/fasta/gallus_gallus/ assessed on 10 February 2022). The RNAfold software of the ViennaRNA package (v1.8.5) (https://www.tbi.univie.ac.at/RNA/#download assessed on 10 February 2022) was used to predict hairpin RNA structures in their flanking sequences.

### 2.4. miRNA Target Gene Prediction and Functional Analysis

In order to detect the differentially expressed miRNAs of shell gland epithelium between the two groups, expression data were analyzed using Log2-transformation. Briefly, the processes were as follows: (1) the level of miRNA was normalized to transcript per million reads (TPM); (2) Qvalue < 0.01 and |log2(foldchange)|  >  1 were made the default thresholds for significantly differential expression.

To elucidate the molecular function of the differentially expressed miRNAs, we used TargetScan to predict their target mRNAs [21]. The target genes were predicted by a custom search using the seed sequences. Before prediction, the 3′ UTR region is required to detect the target site, which is determined conversely for miRNA coherence in multiple animals [22,23]. Using the Database for Annotation, Visualization and Integrated Discovery (DAVID) bioinformatics resources, the genes were classified according to Kyoto Encyclopedia of Genes and Genomes (KEGG) and Gene Ontology (GO) functional annotations to identify pathways that were actively regulated by miRNAs in the shell gland epithelium of green- and light green-egg-laying hens [24].

### 2.5. Quantitative Real-Time PCR

The total RNA was extracted from shell gland epithelium of the two groups, using miRNA 1st Strand cDNA Synthesis Kit and miRNA Universal SYBR qPCR Master Mix (Vazyme, MQ101, Nanjing, Jiangsu, China) to conduct reverse transcription and qRT-PCR. The reactions are as follows: (1) 1 µg total RNA, 2 µL 5 × gDNA wiper Mix, and RNase-free water were mixed and incubated at 42 °C for 2 min. (2) After incubation, 2 µL stem-loop primer (2 µM), 2 µL 10 × RT mix, 2 µL HiScript II Enzyme Mix, and RNase-free water were added into the mixture and subsequently incubated at 25 °C for 5 min, 50 °C for 15 min, and 85 °C for 5 min.

The qRT-PCR mixture contained 2 µL cDNA, 0.4 µL Specific Primer, 0.4 µL mQ Primer R, 10 µL miRNA Universal SYBR qPCR Master Mix, and 7.2 µL RNase-free water. The system was as follows: 95 °C for 5 min, followed by 40 cycles of 95 °C for 10 s, 60 °C for 30 s and 95 °C for 15 s, 60 °C for 60 s as well as 95 °C for 15 s. The cycle threshold (Ct) value was used in the 2-ΔΔCT method to detect each gene’s expression [25]. The designed specified primers are listed in Table S1. The obtained CT values were calculated and graphs drawn using GraphPad Prism (v9.2.0, San Diego, CA, USA).

### 2.6. Statistical Analysis

Interactive and main effects were analyzed using one-way ANOVA with SPSS 20.0 software (SPSS Inc., Chicago, IL, USA). When one-way ANOVA indicated significant differences, these were further explored among individual group means using Fisher’s protected least significant difference test. Differences were considered statistically significant at a *p*-value of less than 0.05. The qRT-PCR data were analyzed using Student’s *t*-test and presented as mean ± standard deviation (SD). Results were deemed statistically significant only if the *p*-value was less than 0.05.

## 3. Results

### 3.1. Eggshell Quality and Phenotypic Results

A total of 2047 eggs were collected on three consecutive days, and ESC, ESI, ESS, EST, and ESW were measured. The L value and a values were used to indicate the intensity of the eggshell greenness, and the lower L and a values were associated with greener eggshells, and vice versa. Although the two groups of chickens used for the miRNA analysis significantly differed in terms of the ESC, they did not differ in terms of other egg parameters (ESI, ESS, EST, and ESW; Table 1 and Figure S1). This sample selection method was applied to eliminate potential interference caused by differences in the other eggshell traits.

### 3.2. Overview of miRNA Sequencing Results

We sequenced the miRNAs from the total RNA samples. A total of 924 miRNAs were identified in the miRNA sequencing results. Among the total miRNAs, there were 587 known miRNAs and 334 novel miRNAs. The target gene results showed that the 924 miRNAs had a total of 22,158 target genes (Table 2).

**Figure 1:**
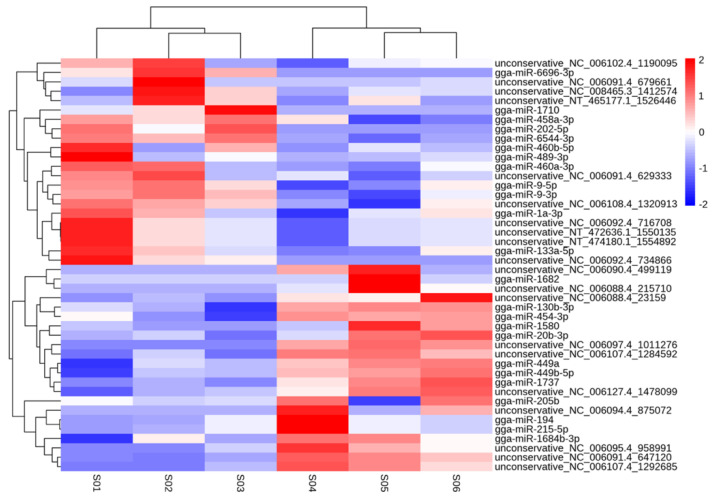
Hierarchical clustering analysis of differentially expressed miRNAs. Clustering of groups based on miRNA expression patterns among groups. S01–S03 were selected from white eggshell groups, and S04–S06 were sorted from green eggshell groups.

To assess the sequencing quality, we analyzed the distribution and length of the miRNAs based on the total abundance and distinct sequences. In the libraries of the two groups, most of the miRNAs were 20–24 nt in size. The most abundant sizes were 22 nt for both the green and white ESCs (Figures S2 and S3). There were 44 differentially expressed miRNAs among the total miRNAs, which comprised 24 known miRNAs and 20 novel miRNAs (Figure S4). The results of the hierarchical clustering analysis of the differentially expressed miRNAs is shown in Figure 1.

### 3.3. Prediction of Target Genes and Functional Bioinformatics Analysis

**Figure 2:**
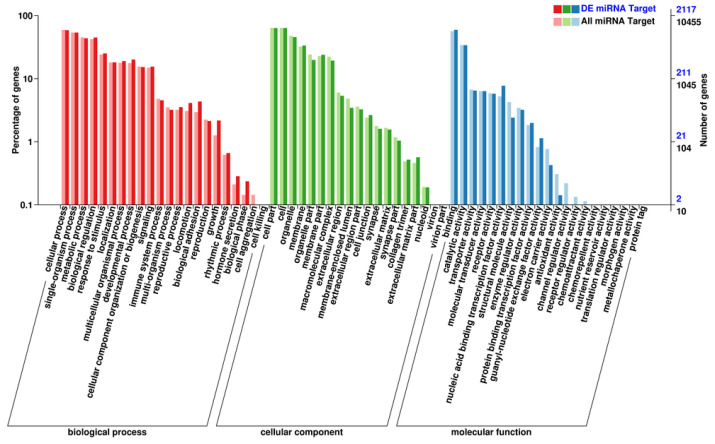
Prediction of target genes and GO functional analysis of differentially expressed miRNAs. The abscissa is GO classification, the left of the ordinate is the percentage of genes, and the right is the number of genes.

The target genes are shown in Supplemental Table S3 and categorized according to their cellular component, molecular function, and biological process (Figure 2). GO enrichment analysis of the target genes showed that the differentially expressed miRNA target genes are mainly related to the functional categories of homophilic cell adhesion, gland development, the Wnt signaling pathway, and epithelial tube morphogenesis (Table S2).

### 3.4. Analysis of KEGG Pathways

**Figure 3:**
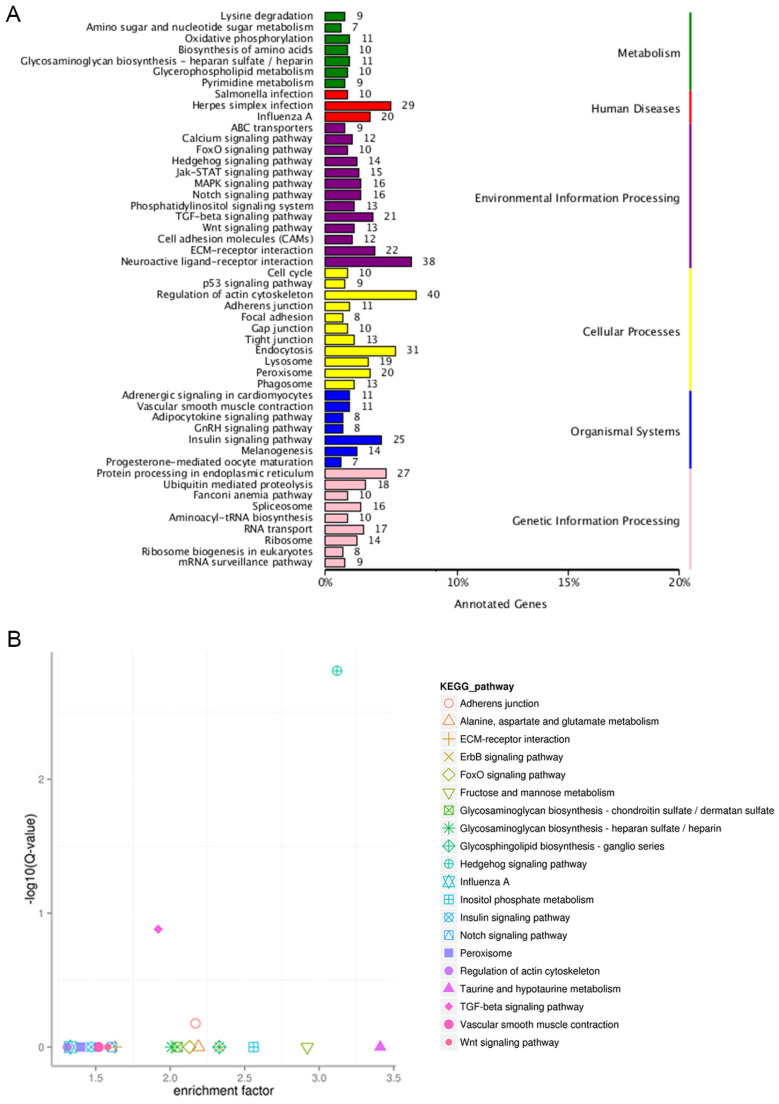
KEGG pathway analysis of differentially expressed miRNAs. (**A**) KEGG annotation graph of differentially expressed miRNA target genes. The ordinate is the name of the KEGG metabolic pathway, and the ordinate is the number of genes annotated with this pathway and their proportion of the total number of genes annotated; (**B**) rich hub diagram of KEGG pathway of differentially expressed miRNA target genes. Each symbol in the figure represents a KEGG pathway, and the pathway name is shown in the legend on the right. The abscissa is the enrichment factor, which represents the proportion of the number of differentially expressed miRNA target genes annotated to a certain pathway to the total number of genes annotated to this pathway.

Using DAVID bioinformatics resources, the predicted target genes were classified according to their KEGG functional annotations (Figure 3A). Interestingly, the differentially expressed miRNA target genes were mainly related to oxidative phosphorylation, neuroactive ligand–receptor interaction, the regulation of the actin cytoskeleton, the insulin signaling pathway, and protein processing in endoplasmic reticulum, while the enrichment analysis showed that only the Hedgehog signaling pathway was significantly transformed in this study (Figure 3B). Although the predicted target genes need to be validated experimentally in subsequent experiments, collectively, the findings illustrate possible pathways and the roles of differentially expressed miRNAs in chickens laying eggs with different shell colors.

### 3.5. Validation of Sequencing

**Figure 4:**
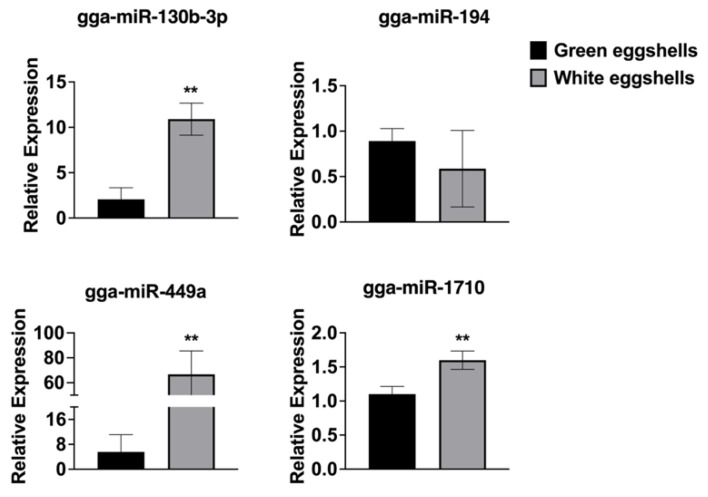
qRT-PCR of differentially expressed miRNAs. The level of detected differential miRNAs was determined by qRT-PCR. ** suggested the *p*-value < 0.01.

Four differential miRNAs were selected randomly to test the reliability of sequencing by qRT-PCR. The qRT-PCR results showed that the levels of miR-310b-3p, miR-449a, and miR-1710 were significantly increased and the expression of miR-194 was decreased (Figure 4), which was consistent with our sequencing results.

## 4. Discussion

The colors of eggshells are generally white, brown, and mottled, and eggshell brownness is common [26]. The intensity of eggshell brownness depends on the concentration of protoporphyrin IX in the eggshell, which is influenced by the total amount of protoporphyrin IX and the size of the eggshell [27]. In contrast, eggshell greenness mainly comprises biliverdin and a small amount of zinc chelate biliverdin [28]. Eggshell greenness is mainly affected by the expression of the oocyan genes (i.e., *SLCO1B3* and *HMOX1*), which are located on the autosomal chromosome 1 [29], while the development of eggshell greenness is due to EAV-HP reversely inserted into the *SLCO1B3* gene [6]. The *SLCO1B3* gene is one of the OATP genes, which are members of the SLC family, and their main function is to transport macromolecules, non-polar drugs, and hormones from the portal vein. This gene also plays an important role in the transport of bile salt compounds, eicosanoids, steroids, and thyroid hormones in certain organisms [30]. The members of the OATP family such as *SLCO1B3*, *SLCO1A2*, *SLCO2B1*, and *SLCO1C1* play an important role in ESC formation by transporting eggshell pigments [31]. The color of eggshells is mainly influenced by genetic factors, while the ability to synthesize protoporphyrin in hens is an important factor that influences the ESC and so is the composition of feed [32]. Some studies have shown that when the content of calcium in the feed increases, calcium deposition in the eggshell will increase, which in turn makes the color of the eggshell lighter and the luster of the eggshell worse [33]. Another study has also found that when the ability to synthesize and secrete calcium is damaged or lost, the ESC fades and becomes white [34]. A recent study also showed that the greenness varies from light to dark, and lncRNAs could partially elucidate this phenomenon [9]. These results indicate that other molecular mechanisms may influence shell greenness rather than just those regulating *SLCO1B3*.

This study found that homozygous green-eggshell hens laid green and white eggs after a peak laying period, suggesting an epigenetic modification in eggshell pigmentation. miRNAs are endogenously expressed RNAs that regulate mRNA expression and have been found to be involved in many biological processes, development, and diseases [35]. It has been reported that miRNAs could regulate the expression of genes related to lipid metabolism and fat deposition to further improve growth performance and meat quality [36]. Early and later feathering is widely employed in determining chicken gender and is attributed to tandem duplication between the *PPLR* and *SPEF2* genes. On several occasions, chicks have shown inconspicuous feather differences after hatching; the latest study found that 14 pairs of miRNA-mRNA perform a negative function on feather formation [36,37]. Ca2+ transport is an essential biological process for eggshell calcification and it has been shown that miR-449c-5p could reduce the expression of *ATP2B4* to promote uterine Ca2+ transport [38]. Therefore, this study aimed to explore the functions of miRNA on the pigmentation of eggshells.

Our study identified multiple differentially expressed known miRNAs (e.g., miR-449a, miR-130b-3p, miR-215-5p, miR-1a-3p, let-7a-3p, and let-7a-5p) that might be relevant to the formation of eggshell greenness. At present, the identification of miRNAs that influence the ESC has rarely been reported. In this study, the expression level of gga-miR-449a significantly differed between the two experimental groups, suggesting that miR-449a may be involved in pigment formation. gga-miR-144-3p could target the *ABCG2* gene, and the present study observed that miR-144-3p expression differed between the two experimental groups. In addition, we also found that differentially expressed miRNAs target *KIT* and *HMOX2* genes. Mutations in Kit have been shown to influence melanin accumulation in the hair follicles and eventually results in white coat color in Yorkshire pigs [39], and Hmox2 could affect biliverdin synthesis [40]. Changes in these genes may jointly contribute to the formation of green eggshells. The KEGG enrichment analysis indicated that the target genes of differentially expressed miRNAs enriched the functional categories of the regulation of the actin cytoskeleton and ABC transporters (Figure 3). In addition, the most representative pathways that were related to the ESC involved ABC transporters, followed by the regulation of the actin cytoskeleton and the PPAR signaling pathway [41,42]. These results suggest that miRNA can target *ABCG2*, *KIT*, and *HMOX2* thus playing a role in the formation of the ESC. Another differentially expressed miRNA between green shell and white shell glands is gga-let-7a-3p, which could change the *SLC5A11* gene expression, and proves that let-7 indeed influences eggshell color development.

Heme oxygenase (HO) was first described as an enzyme that catalyzes the degradation of heme to biliverdin [43]. HO-1 is a key enzyme for the formation of biliverdin, and biliverdin is one of the important pigments that is responsible for the ESC. Related studies have shown that the formation of eggshell pigments is determined by several genes [44,45]. Wang et al. found that the HO-1 gene plays an important role in the formation of eggshell biliverdin and could lead to blue egg formation when it is upregulated [46]. Recent studies have shown that the HO-1 gene plays an important role in the antioxidant process, indicating that this gene may be involved in the regulatory mechanism of eggshell greenness by inhibiting or activating certain related biological processes [47,48]. In the present study, a GO enrichment analysis identically showed that the target genes and differentially expressed miRNAs enriched the functional category of antioxidant activity (Figure 2). Furthermore, we identified the Hedgehog signaling pathway as the significantly enriched pathway, which proved an indispensable function in retinal pigment epithelium differentiation [49]. A later study suggested that the Hedgehog signaling pathway could affect normal pigmentation in retinal pigment epithelium [50]. The Hedgehog signaling pathway may change the pigment accumulation on the shell gland epithelium and further increase the color diversity of eggshells. These findings could be employed to improve the breeding process, increase the green egg rate, and support economic benefits.

## 5. Conclusions

The formation of green eggs results from an avian endogenous retrovirus fragment inserted into the *SLCO1B3* gene. However, various egg colors have been observed in poultry production, suggesting a potential epigenetic regulatory mechanism. This study is the first investigation on miRNAs that are related to the formation of a green ESC. We identified 44 differentially expressed miRNAs that are related to ESC formation in chickens, which target hundreds of genes, including *ABCG2*, *KIT*, and *HMOX2*, and multiple solute carrier family genes. The functional analysis indicates that ABC transporters, the SLC family, and the Hedgehog signaling pathway may influence the transport process of pigment precursors, regulating the formation of eggshell pigmentation. These findings suggest a novel mechanism to further explore the pigmentation of eggshells in birds and can be employed to promote poultry industry development in the future.

## Figures and Tables

**Table 1:** Comparison of eggshell quality between the two groups of chickens.

Eggshell Quality	Light Green Eggshell Group (N = 3)	Dark Green Eggshell Group (N = 3)	Population (N = 856)
Egg weight (g)	40.13 ± 1.91	39.03 ± 3.09	39.18 ± 4.21
ESW (g)	5.76 ± 0.26	5.63 ± 0.41	5.46 ± 0.56
ESI	1.31 ± 0.04	1.32 ± 0.04	1.31 ± 0.04
ESC (L value)	86.33 ± 1.29 **	79.79 ± 1.69 **	82.93 ± 1.79 **
ESC (a value)	−2.5 ± 0.87 *	−6.3 ± 0.85 *	−4.07 ± 1.19 *
ESC (b value)	5.72 ± 1.77	6.03 ± 1.31	5.97 ± 1.33
Eggshell glossiness (Gu)	5.83 ± 1.48	6.17 ± 3.40	5.96 ± 2.32
ESS (kg/cm^2^)	3.618 ± 0.769	3.638 ± 0.774	3.643 ± 0.838
EST (mm)	0.388 ± 0.04	0.400 ± 0.034	0.393 ± 0.043

N—number of chickens; ESW—eggshell weight; ESI—egg shape index; ESC—eggshell color; ESS—eggshell strength; EST—eggshell thickness; ** *p* < 0.01; * *p* < 0.05.

**Table 2:** Number of miRNAs and their target genes.

MiRNA Type	Total Number of miRNAs	Number of miRNAs with Target Genes ^1^	Number of Target Genes ^2^
Known_miRNAs	587	499	20,890
Novel_miRNAs	334	304	19,893
Total	921	803	22,158

^1^ miRNA with target: number of miRNAs predicted to target genes. ^2^ Target gene: predicted number of target genes.

## Data Availability

The miRNA sequencing data from this study are openly available through NCBI (https://www.ncbi.nlm.nih.gov assessed on 10 February 2022) under Bioproject accession number PRJNA856885.

## Supplementary Materials

The following supporting information can be downloaded at: https://www.mdpi.com/article/10.3390/genes15060811/s1, Figure S1: Histogram of eight phenotypes from chicken populations; Figure S2: Size distribution of miRNAs in green eggshell; Figure S3: Size distribution of miRNAs in white eggshell; Figure S4: Volcano map of the total miRNAs; Table S1: Primers sequences for qRT-PCR; Table S2: List of significantly enriched GO terms.
