# Supplementary material for: Identification and Characterization of the miRNA Transcriptome Controlling Green Pigmentation of Chicken Eggshells
Source: Genes (Basel). 2024 Jun 19;15(6):811. doi: 10.3390/genes15060811 (PMC11202967; doi:10.3390/genes15060811)
Supplement: Supplementary file 1 [file genes-15-00811-s001.zip › Supplemental figures.pdf]

Supplemental Figure S1. Histogram of eight phenotypes from chicken populations.

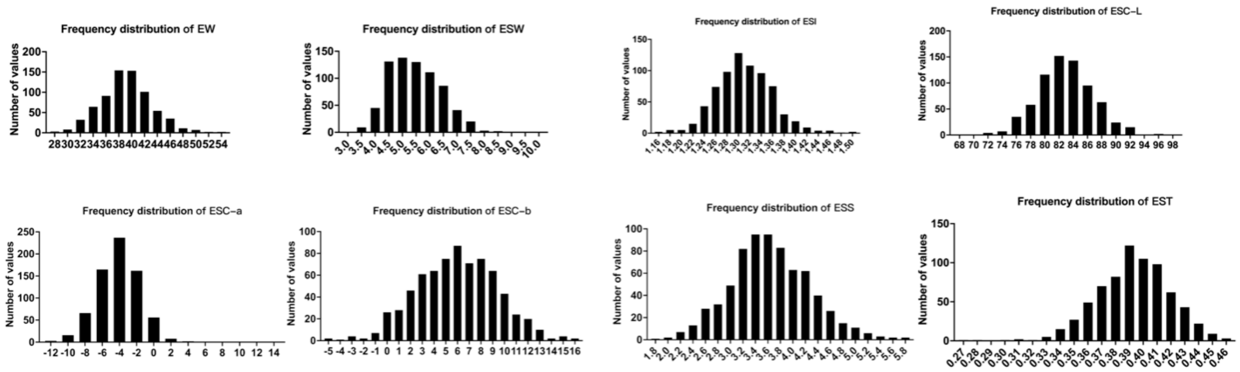

EW, egg weight; ESW, eggshell weight; ESI, egg shape index; ESC-L, eggshell color-L value; ESC-a, eggshell color-a value; ESC-b, eggshell color-b value; ESS, eggshell strength; EST, eggshell thickness.

Supplemental Figure S2. Size distribution of miRNAs in green eggshells.

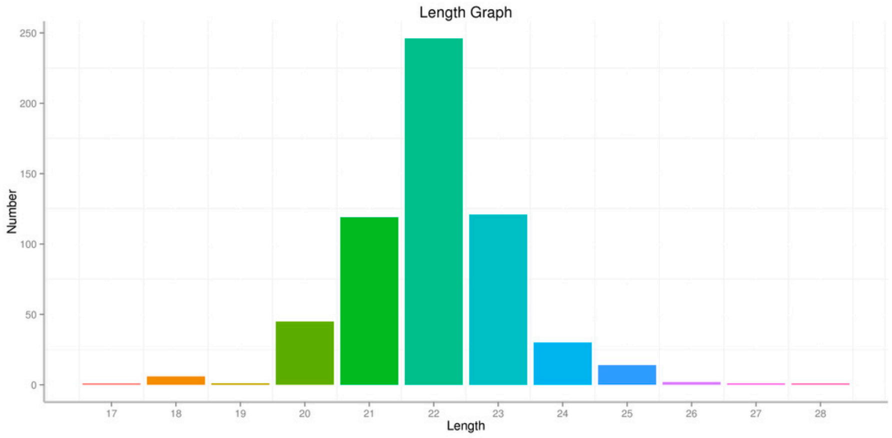

Length distribution graph of known miRNA. The abscissa is the miRNA length, and the ordinate is the number of miRNAs with a specific length.

Supplemental Figure S3. Size distribution of miRNAs in white eggshells.

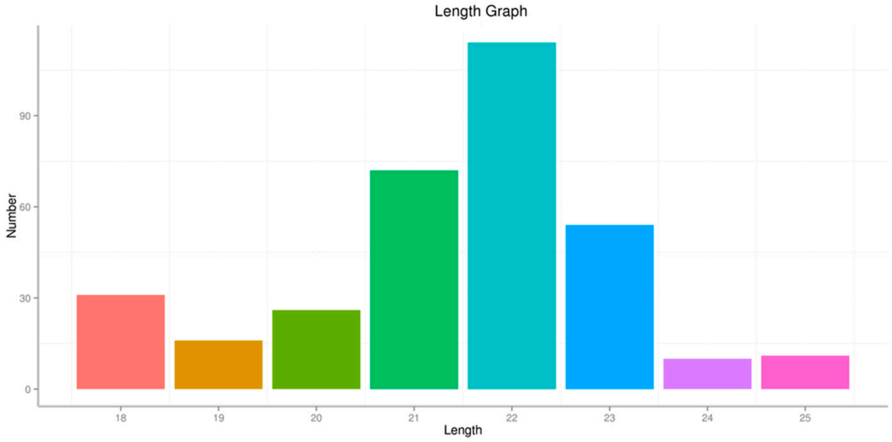

Length distribution graph of predicted miRNA. The abscissa indicates miRNA length and the ordinate represents the number of miRNAs with specific length

Supplemental Figure S4. Volcano map of the total miRNAs.

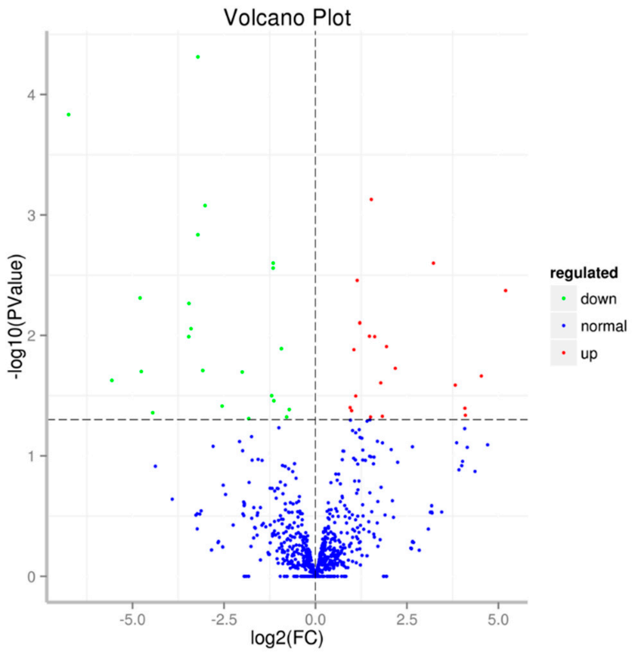

The volcano plot suggest differentially expressed genes in shell gland epithelium. Red dots indicate up-regulated miRNAs and green dots represent down-regulaated.
